# The *Escherichia coli* two-component signal sensor BarA binds protonated acetate *via* a conserved hydrophobic-binding pocket

**DOI:** 10.1016/j.jbc.2021.101383

**Published:** 2021-11-04

**Authors:** Adrián F. Alvarez, Claudia Rodríguez, Ricardo González-Chávez, Dimitris Georgellis

**Affiliations:** Departamento de Genética Molecular, Instituto de Fisiología Celular, Universidad Nacional Autónoma de México, México City, México

**Keywords:** two-component system, BarA sensor kinase, sensor domain, stimulus, signal reception, Cache, calcium channels and chemotaxis receptors, PAS, Per-Arnt-Sim, PD, periplasmic domain, TCS, two-component system, TM, transmembrane

## Abstract

The BarA/UvrY two-component signal transduction system is widely conserved in γ-proteobacteria and provides a link between the metabolic state of the cells and the Csr posttranscriptional regulatory system. In *Escherichia coli*, the BarA/UvrY system responds to the presence of acetate and other short-chain carboxylic acids by activating transcription of the noncoding RNAs, CsrB and CsrC, which sequester the RNA-binding protein CsrA, a global regulator of gene expression. However, the state of the carboxyl group in the acetate molecule, which serves as the BarA stimulus, and the signal reception site of BarA remain unknown. In this study, we show that the deletion or replacement of the periplasmic domain of BarA and also the substitution of certain hydroxylated and hydrophobic amino acid residues in this region, result in a sensor kinase that remains unresponsive to its physiological stimulus, demonstrating that the periplasmic region of BarA constitutes a functional detector domain. Moreover, we provide evidence that the protonated state of acetate or formate serves as the physiological stimulus of BarA. In addition, modeling of the BarA sensor domain and prediction of the signal-binding site, by blind molecular docking, revealed a calcium channels and chemotaxis receptors domain with a conserved binding pocket, which comprised uncharged polar and hydrophobic amino acid residues. Based on the comparative sequence and phylogenetic analyses, we propose that, at least, two types of BarA orthologues diverged and evolved separately to acquire distinct signal-binding properties, illustrating the wide adaptability of the bacterial sensor kinase proteins.

The BarA/UvrY two-component system (TCS) is a central element for the modulation of the Csr posttranscriptional regulatory system that allows γ-proteobacteria, such as *Escherichia coli*, to coordinate numerous physiological processes, including carbon metabolism, motility, biofilm formation, peptide uptake, and virulence (1, 2). This TCS comprises the transmembrane sensor histidine kinase BarA, which belongs to the subclass of hybrid-tripartite histidine kinases (3, 4) and its cognate response regulator UvrY (5). Upon signal perception, BarA autophosphorylates at the expense of ATP and transphosphorylates the response regulator UvrY (5, 6). The phosphorylated form of UvrY (UvrY-P), in turn, activates the transcription of the CsrB and CsrC noncoding RNAs (2, 7), which possess repeated sequence elements that allow them to interact with multiple molecules of the CsrA protein and prevent its interaction with mRNA targets (8, 9). CsrA is an RNA-binding protein that directly interacts with the 5′ untranslated leaders of mRNA targets and controls gene expression by regulating their translation, stability, and/or elongation (10, 11, 12, 13, 14).

In an earlier study, we reported that the *E. coli* BarA protein senses the presence of acetate (15), which accumulates in the growth media as the cells metabolize acetogenic substrates, such as glucose (16), or other short-chain carboxylic acids, such as formate and propionate (15). However, the region of BarA that detects the physiological stimulus has not yet been identified. Nevertheless, the sensing domain of the BarA ortholog protein in *Pseudomonas aeruginosa*, GacS, was proposed to be in its periplasmic domain (PD). The structure of this domain was recently solved (17), revealing that it relies on a PhoQ, DcuS, CitA (PDC)/Per-ARNT-Sim (PAS)-like folding that includes an essential positively charged pocket, which was proposed to accommodate a negatively charged ligand. The PAS domains are widely distributed modules that are involved in sensing environmental and nutritional signals, such as metabolites, gases, light or redox potential (18, 19, 20) and are characterized by a conserved β-sheet core flanked by α-helices and loops that provide ligand-binding specificity (18, 21). PDC/PAS-like domains are extracytoplasmic domains (22, 23, 24) and are often found as signal-recognition modules of sensor kinases or chemoreceptors (25). The PDC/PAS-like domains, which are closely related to the PAS superfamily, belong to the Cache (calcium channels and chemotaxis receptors) sensory domain superfamily (26). Various Cache sensors that detect carboxylate-harboring signals have been functionally and/or structurally characterized, and it has been demonstrated that all of them interact with the ligand in its anionic state through the formation of salt bridges with basic residues that constitute a positively charged pocket (17, 21, 22, 24, 27, 28, 29, 30).

The present work aims to elucidate the role of the periplasmic region of the BarA HK in signal perception, by genetic, biochemical, and bioinformatic methods. We show that the PD of BarA is needed for proper switching between its phosphatase and kinase activities in response to the physiological stimulus and thereby for regulating the activity of the BarA/UvrY-signaling pathway. Moreover, we demonstrate that the putative signal-binding pocket does not rely on positively charged residues but instead on uncharged polar and hydrophobic residues. We, also, provide evidence that the protonated state of formic acid and acetic acid constitute the physiological stimuli for the BarA sensor kinase. Finally, our phylogenetic analysis of the PD of BarA ortholog proteins revealed that they could be grouped into two classes depending on the amino acid residue conservation pattern of the putative ligand-binding site, suggesting that the members of these two BarA groups have evolved to perceive different stimuli.

## Results and discussion

### The periplasmic region of BarA is required for activation of its kinase activity

**Figure 1:**
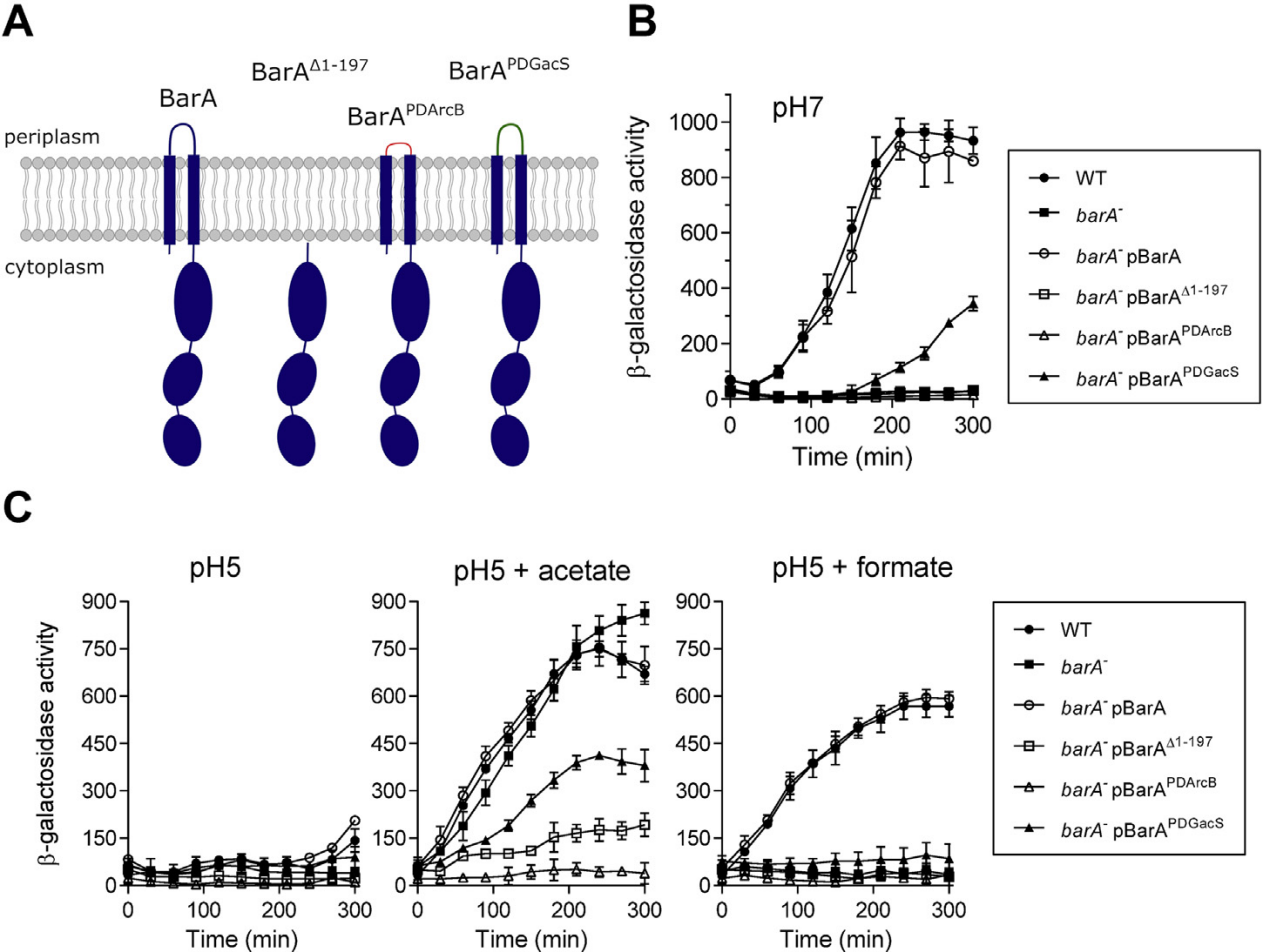
**Testing the importance of the BarA periplasmic domain.***A*, schematic representation of the BarA HK and the truncated or chimeric BarA proteins used in this study. *B*, overnight cultures of the isogenic strains KSB837 (WT) (*filled circles*), IFC5036 (*barA*^−^) (*filled squares*), and IFC5036 (*barA*^−^) carrying plasmid pEXT22-barA (pBarA) (*open circles*), pEXT22-barAcyt (pBarA^Δ1–197^) (*open squares*), pEXT22-barAPDarcB (pBarA^PDArcB^) (*open triangles*), or pEXT22-barAPDgacS (pBarA^PDGacS^) (*filled triangles*), were diluted to an A_600_ of 0.001 in LB medium. When the cultures reached an A_600_ of 0.15, the β-galactosidase activity was followed for 300 min. *C*, the cultures of the same strains described for (*B*) were grown in LB medium, the pH of which had been adjusted and buffered to 5.0. At an A_600_ of 0.15, a sample was withdrawn (time = 0 min), and the culture was divided into three parts: one of them continued its incubation without any supplement (*left panel*), whereas acetate (7 mM) (*central panel*) or formate (7 mM) (*right panel*) was added to the other cultures, and the β-galactosidase activity was followed. The averages from three independent experiments are presented, and the SDs (*error bars*) are indicated. PD, periplasmic domain.

It has been previously reported that short-chain carboxylic acids, such as formate and acetate, act as the physiological stimuli for BarA (15). However, the BarA signal reception site remains elusive. Because the periplasmic region of most sensor kinases constitutes the sensor domain, we explored the functionality of the periplasmic segment of BarA. To this end, a low-copy number plasmid expressing the cytosolic part of the BarA protein (BarA^Δ1–197^), lacking the transmembrane (TM) and the periplasmic domains, and two plasmids carrying *barA* alleles in which the periplasmic segment of the protein was replaced by the corresponding section of either the *E. coli* ArcB HK (BarA^PDArcB^) or the *Azotobacter vinelandii* GacS HK (BarA^PDGacS^) were constructed (Fig. 1*A*). ArcB was chosen because its periplasmic bridge is unusually short (16 aa residues), and it does not participate directly in the reception of any signal (31), whereas GacS is the BarA homolog of Pseudomonads, the activating signal of which remains unknown. The above constructed plasmids were transformed into the Δ*barA* strain IFC5036, carrying a λφ(*csrB-lacZ*) fusion, and the activity of the BarA variants was analyzed by monitoring the *in vivo* levels of phosphorylated UvrY, as indicated by the expression of the *csrB-lacZ* reporter, which depends directly on the activity of the BarA/UvrY TCS (2). The transformants were grown in LB medium buffered at pH 7.0, and the *csrB-lacZ* expression was monitored. As to be expected, reporter expression was activated at the transition from exponential to stationary phase of growth in both the WT strain and the *barA* mutant harboring a plasmid-borne WT *barA* allele, whereas no activation of reporter expression was observed in the *barA*^−^ strain (Fig. 1*B*). Interestingly, the construct where the BarA PD was replaced by the corresponding GacS region resulted in a delayed increase of reporter expression, reaching ∼30% of the WT expression values (Fig. 1*B*). Thus, the PD of GacS may be able to respond to acetate, although less efficiently than the corresponding region of BarA, or it responds to another stimulus that is present at the transition from exponential to the stationary phase of growth. However, the possibility that structural distortions of the BarA-GacS hybrid do not allow its proper activation cannot be discarded. On the other hand, when the BarA TM and periplasmic domains were removed, or when the BarA periplasmic bridge was replaced by the ArcB counterpart, no activation of the *csrB-lacZ* expression was noted (Fig. 1*B*), suggesting that either these BarA variants are inactive proteins, or that they are unable to switch from their phosphatase to their kinase activity upon entry into the stationary phase of growth. To confirm that each BarA-hybrid variant remains functional, we evaluated the *csrB-lacZ* expression in the above strains grown in LB buffered at pH 5.0. Such a condition provides an environment that does not allow the activation of BarA (32) unless acetate or formate is added to the growth medium (15). It is relevant to mention that both acetate and formate act through BarA, whereas acetate can also activate *csrB* expression by promoting the acetyl-P-dependent phosphorylation of UvrY in the absence of BarA (15). This fact permits the evaluation of both the kinase and the phosphatase activities of BarA and its variants (33). As expected, no activation of the *csrB-lacZ* expression was observed in any of the tested strains at pH 5.0 (Fig. 1*C*), whereas addition of acetate or formate to the growth medium resulted in the immediate activation of the reporter expression in the WT strain and in the *barA* mutant harboring a plasmid-borne WT *barA* allele (Fig. 1*C*). Also, addition of acetate, but not formate, resulted in the activation of reporter expression in the Δ*barA* mutant strain (Fig. 1*C*), due to acetyl-P-dependent UvrY phosphorylation, in agreement with previous reports (15, 34). On the other hand, no activation of the *csrB-lacZ* expression was observed in the strains expressing the BarA^Δ1–197^ or the BarA^PDArcB^ proteins, indicating that these BarA variants fail to respond to acetate and formate but retain their phosphatase activity and are therefore functional proteins that remain locked in their phosphatase state. Moreover, activation of the *csrB-lacZ* expression was slower and reduced by about 2-fold in the BarA^PDGacS^ expressing strain upon acetate addition, whereas no activation of reporter expression was observed after formate addition (Fig. 1*C*). Thus, the BarA^PDGacS^ chimeric HK appears to comprise an impaired phosphatase activity. Alternatively, it may perceive a stimulus (other than acetate) whose production increases at pH 5.0 in the presence of acetate and is also produced at pH 7.0 in the stationary phase of growth. This is consistent with previous reports in which soluble metabolic molecules produced at the end of the exponential growth, such as tricarboxylic acid cycle intermediates, were proposed to activate the GacS sensor kinase (35, 36). Nevertheless, the above results demonstrate that the PD of the *E. coli* BarA is required for proper switching between its phosphatase and kinase activities in response to the physiological stimulus.

### Structural modeling of the BarA periplasmic domain and identification of putative signal-binding residues

**Figure 2:**
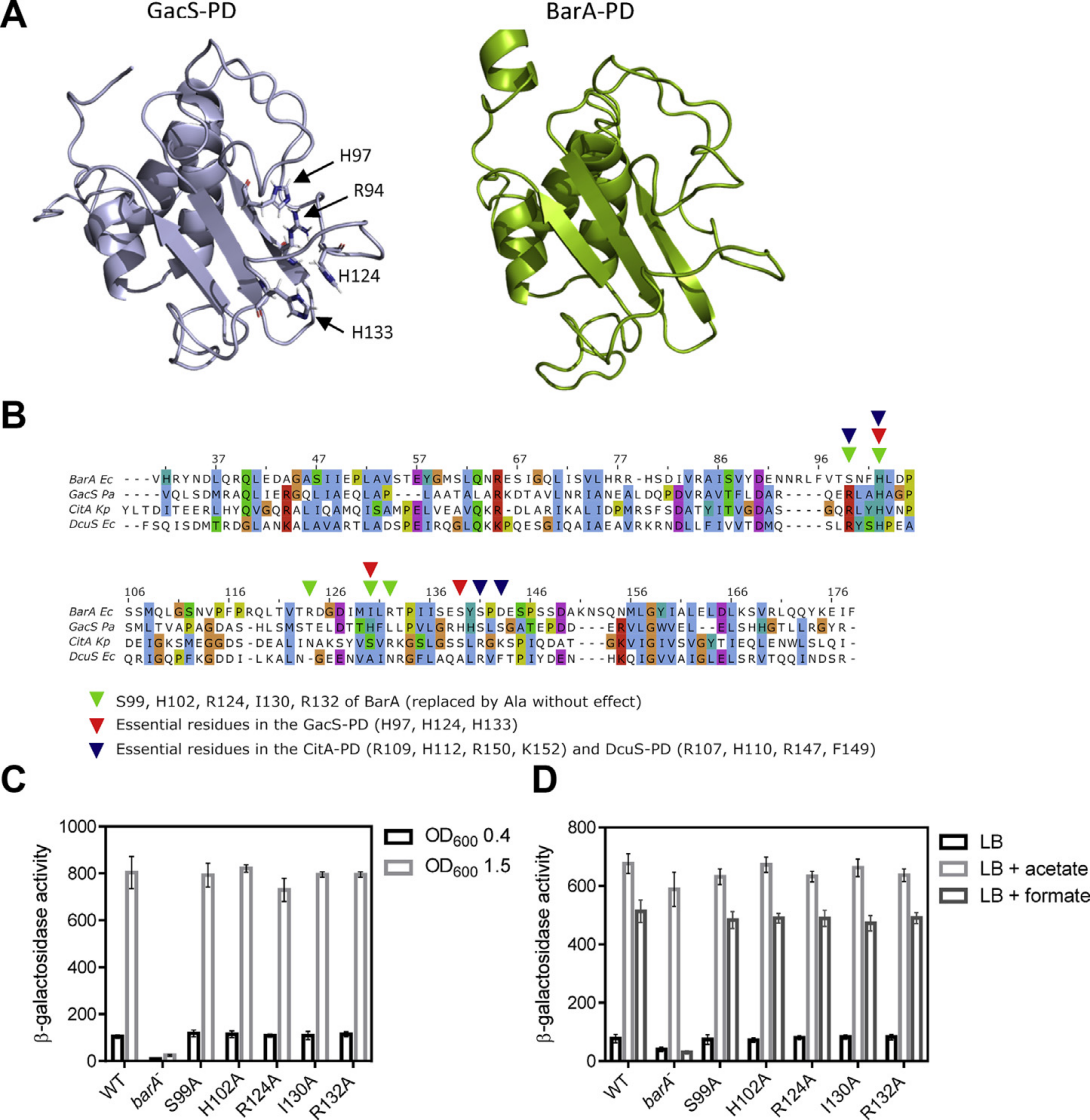
**Analysis of conserved residues and structural model of the BarA periplasmic domain.***A*, superposed solution structure of the GacS sensor domain (*left*) and predicted structure for the BarA-PD (*right*). The 3D structural model of BarA-PD was generated by using the program I-Tasser. The relative position of conserved residues involved in GacS signaling (17) is indicated. *B*, sequence alignment of the BarA-PD with related PDC/PAS-like containing sensor domains. The Clustal X color-scheme was used to visualize the residue conservation patterns. The numbers at the top of the alignment indicate the amino acid positions in full-length BarA. The position of essential residues in the GacS or in the CitA/DcuS sensor domains are indicated by *red triangles* or by *blue triangles*, respectively. The residues replaced by Ala in the BarA-PD are indicated by *green triangles*. *C*, the strains KSB837 (WT), IFC5035 (*barA*^−^), IFC5038 (*barA*^S99A^), IFC5039 (*barA*^H102A^), IFC5040 (*barA*^R124A^), IFC5041 (*barA*^I130A^), and IFC5042 (*barA*^R132A^) were grown in LB medium to an A_600_ of 0.4 (nonstimulatory conditions, *black bars*) or 1.5 (stimulatory conditions, *gray bars*), and the β-galactosidase activity was measured. The average from three independent experiments is presented, and the SDs (*error bars*) are indicated. *D*, the strains KSB837 (WT), IFC5035 (*barA*^−^), IFC5038 (*barA*^S99A^), IFC5039 (*barA*^H102A^), IFC5040 (*barA*^R124A^), IFC5041 (*barA*^I130A^), and IFC5042 (*barA*^R132A^) were grown in LB medium buffered at pH 5.0. At an A_600_ of 0.15, the culture was split in three, and 7 mM acetate (light *light-gray bars*) or formate (*dark-gray bars*) was added to two of them, whereas the third one was used as a control (*black bars*), and the cultures were incubated for 180 min before the samples were withdrawn for β-galactosidase quantification. The average and SDs (*error bars*) from three independent experiments are shown. Ec, *Escherichia coli*; I-Tasser, Iterative Threading Assembly Refinement; Kp*, Klebsiella pneumoniae*; Pa, *Pseudomonas aeruginosa*; PD, periplasmic domain.

To explore the structural features of the putative BarA sensor domain, we generated a structural model of the BarA PD (residues 31–178 of BarA), based on the NMR structure of the *P. aeruginosa* GacS-PD (Protein Data Bank code: 5O7J) (17), using the Iterative Threading Assembly Refinement server (37) (Fig. 2*A*). The model shows the typical architecture of a group of signal-recognition domains that adopt a particular α/β fold known as PDC/PAS-like domain (Fig. 2*A*), initially described for the sensor domains of the PhoQ, DcuS, and CitA histidine kinases (22, 23, 24). The PDC/PAS-like domain, currently regarded as Cache domain (26), is characterized by a central three-to-five antiparallel β-sheet flanked by an N-terminal α-helix and additional loops and helices on each side (25). To identify residues implicated in signal sensing, we performed a comparative sequence analysis of the BarA-PD with the sensor domains of GacS, DcuS, and CitA, all of which contain well characterized positively charged binding pockets that allow the interaction with negatively charged ligands (Fig. 2*B*). The ligand binding pockets include residues Arg94, His97, His124, and His133 in GacS; Arg107, His110, and Arg147 in DcuS; and Arg109, His112, Arg150, and Lys152 in CitA. From these basic amino acid residues, the only one that appears to be conserved in the BarA-PD sequence is His102 (corresponding to His97 of GacS, His110 of DcuS, and His112 of CitA), whereas Ser99 seems to be at the position of the conserved Arg residue present in GacS (Arg94), DcuS (Arg107), and CitA (Arg109). It is important to mention that this pair of basic residues was shown to be essential for signal binding in DcuS and CitA (30, 38), whereas only His97 was needed for GacS signaling (17). Curiously, the other residues that constitute the positively charged pocket required for ligand binding in GacS (17), DcuS (21, 30) and CitA (38), were not found in the BarA sequence. Nevertheless, based on their positive charge and/or their relative position in the sequence alignment with known PDC/PAS-like sensor domains, the residues Ser99, His102, Arg124, Ile130, and Arg132 of BarA were selected to test whether they could be involved in signal reception. To this end, five strains carrying the *barA*^S99A^, *barA*^H102A^, *barA*^R124A^, *barA*^I130A^, or *barA*^R132A^ mutant allele and the λφ(*csrB-lacZ*) transcriptional fusion were generated. Alanine was chosen because it is uncharged, and it is not bulky enough to cause major structural distortions in the putative-binding pocket. The generated mutant strains, IFC5038 (*barA*^S99A^), IFC5039 (*barA*^H102A^), IFC5040 (*barA*^R124A^), IFC5041 (*barA*^I130A^), and IFC5042 (*barA*^R132A^) along the WT and Δ*barA* strains were grown in LB buffered at pH 7.0 to an absorbance at 600 nm (A_600_) of 0.4 (nonstimulatory condition) or to an A_600_ of 1.5 (stimulatory condition), and the *csrB-lacZ* reporter expression was measured. It was found that substitution of Ser99, His102, Arg124, Ile130, or Arg132 to Ala did not affect reporter expression (Fig. 2*C*). Next, to test whether these substitutions affect the BarA phosphatase activity, the expression of the *csrB-lacZ* reporter was measured in the WT and mutant strains grown at pH 5.0 in the presence or the absence of either acetate or formate (Fig. 2*D*). No difference between the WT and the five BarA mutant variants was noted, indicating that none of the above amino acid residues is essential for BarA signal reception. In addition, these results suggest that the BarA sensor domain does not rely on a positively charged ligand-binding pocket, providing an important difference between the BarA sensor domain and other structural related sensor domains, including the one of the *P. aeruginosa* GacS.

### Identification of amino acid residues required for proper BarA signaling

**Figure 3:**
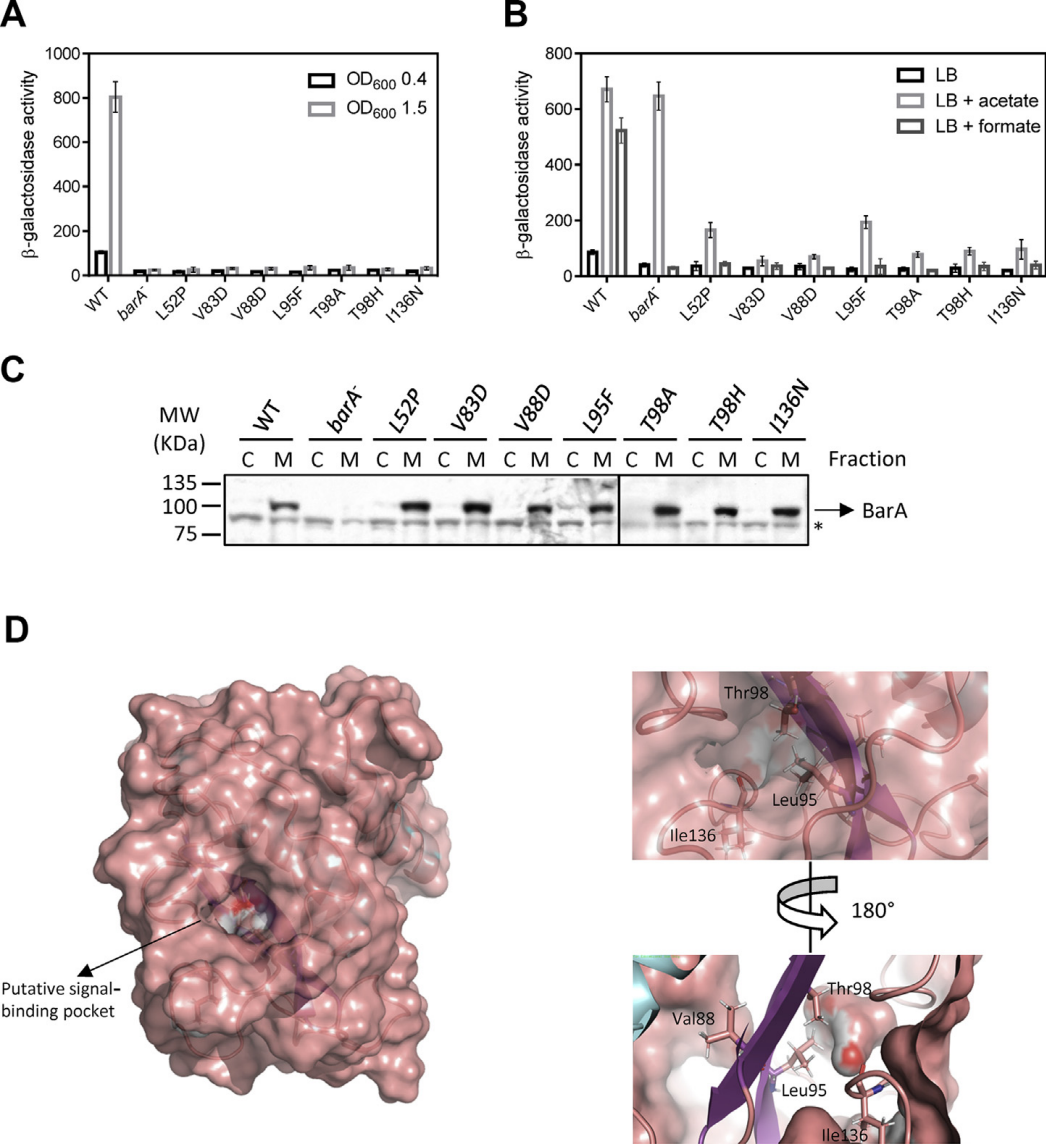
**Identification and spatial disposition of essential residues in the BarA sensor domain.***A*, the cells of strains KSB837 (WT), IFC5035 (*barA*^−^), and IFC5035 carrying pACYCDuet-1 derivative plasmids that harbor the *barA* mutant variants were grown in LB buffered at pH 7.0 to midexponential growth phase (absorbance _∼_0.4) (nonstimulatory conditions, *black bars*) or to an A_600_ of 1.5 (stimulatory conditions, *gray bars*) and the β-galactosidase activity was measured. *B*, the strains KSB837 (WT), IFC5035 (*barA*^−^), and IFC5035 carrying pACYCDuet-1 derivative plasmids harboring the *barA* mutant variants were grown in LB medium buffered at pH 5.0; at an A_600_ of 0.15, the culture was split in three, and 7 mM acetate (*light-gray bars*) or formate (*dark-gray bars*) was added to two of them, whereas the third one was used as a control (*black bars*), and the samples for β-galactosidase quantification were withdrawn after 180 min. In (*A*) and (*B*), the average from three independent experiments is presented, and the SDs (*error bars*) are indicated. *C*, the BarA protein (102.5 KDa) levels in cytosolic (C) and membrane (M) fractions of strains KSB837 (WT), IFC5035 (*barA*^−^), and IFC5035 carrying pACYCDuet-1 derivative plasmids harboring the *barA* mutant variants, as determined by Western blot analyses using BarA polyclonal antibodies. ∗ indicates a nonspecific signal that was observed in all stains and fractions. *D*, a surface model depicting the predicted BarA signal-binding pocket. The whole view of the BarA-PD (*left*) and zoomed-in views of the putative binding pocket (*right*) are presented. The residues Leu95, Thr98 ,and I136 required for BarA signaling are indicated.

Attempts to identify specific amino acid residues within the BarA periplasmic portion that may participate in signal reception, by an unbiased approach, were then pursued. The DNA region codifying the periplasmic portion of BarA was mutagenized by error-prone PCR, and the PCR products were used to replace the corresponding region of the *barA* gene that was previously cloned into the pACYCDuet-1 plasmid. In this construct, *barA* is under the control of a T7 promoter, whose basal activity results in nearly WT BarA levels, as indicated by Western blot analysis (Fig. S1). The pool of plasmids was first screened for loss of kinase activity mutants, by streaking individual colonies onto LB agar at pH 7.0 supplemented with X-gal and selecting white or light-blue colonies. The selected clones were further screened for phosphatase active *barA* alleles by streaking them onto LB agar at pH 5.0 supplemented with X-gal and 7 mM acetate and selecting white or light-blue colonies. Sequencing of the selected clones identified seven alleles harboring single-point missense mutations, namely *barA*^L52P^, *barA*^V83D^, *barA*^V88D^, *barA*^L95F^, *barA*^T98A^, *barA*^T98H^, or *barA*^I136N^. To validate the kinase activity of these mutant *barA* alleles, the cells of WT, Δ*barA*, and Δ*barA* harboring either of the seven mutant plasmid-borne *barA* alleles were grown in LB buffered at pH 7.0 to nonstimulatory (A_600_ of 0.4) or stimulatory (A_600_ of 1.5) conditions, and the *csrB-lacZ* reporter expression was measured. It was found that the *csrB-lacZ* reporter activation under stimulatory conditions occurred only in the WT but not in the Δ*barA* or the Δ*barA* harboring any of the seven mutant plasmid-borne *barA* alleles (Fig. 3*A*). To verify that the mutant BarA variants are functional proteins, the strains carrying the WT or mutant BarA alleles were grown at pH 5.0, and their *csrB-lacZ* expression was measured in the presence or the absence of acetate or formate (Fig. 3*B*). As anticipated, no activation of reporter expression was observed in any of the tested strains in the absence of acetate or formate, whereas the *csrB-lacZ* expression increased in the Δ*barA* strain only in the presence of acetate, due to acetyl-P dependent UvrY phosphorylation (Fig. 3*B*). On the other hand, in contrast to the WT strain, where the *csrB-lacZ* expression was activated in the presence of acetate or formate, the seven-BarA mutants were unable to respond to formate. In addition, in the presence of acetate, the V83D, V88D, T98F, T98H, and I136N BarA mutants showed a 7- to 10-fold lower *csrB-lacZ* expression than the WT or the Δ*barA* strain, whereas the L52P or L95F BarA variants showed an almost 4-fold lower *csrB-lacZ* expression. To ascertain that each BarA mutant protein is properly expressed and remains membrane associated, we performed Western blot analyses on cytosolic and membrane fractions of the cells. In all cases, the mutant proteins were found to be expressed at nearly WT BarA levels and were associated with the cytoplasmic membrane (Fig. 3*C*). It can, therefore, be concluded that the selected BarA mutants are functional proteins that retain their UvrY-P phosphatase activity despite the presence of the stimulus. Next, we examined the relative position of the identified residues in the above-presented structural model of BarA-PD. It was noted that Leu95 and Thr98 are located in the second β strand, overlapping the position of Arg94 and His97 in the GacS-PD structure, whereas Ile136 is predicted to be located in the major loop between the second and the third β strand (Fig. 3*D*), and the side chains of these three amino acid residues appear to occupy the putative ligand-binding pocket (Fig. 3*D*), suggesting that they could be directly involved in signal binding. On the other hand, Val83 and Val88 are located in the first β strand but with their hydrophobic side chains facing to the opposite side with respect to the predicted signal binding site. The fact that the replacement of these two residues by Asp in the BarA protein resulted in a constitutive phosphatase phenotype could be explained by structural distortions caused by the negative-charged aspartate residue that may impact the conformation of the binding pocket. However, the possibility that the true orientation of these residues differs from the one predicted by the model cannot be excluded. In addition, the predicted position of Leu52 falls outside the putative ligand binding-pocket and would not be expected to be directly involved in acetate binding. Instead, the substitution of Leu52 by Pro may give rise to structural distortions, which affect the orientation of the whole periplasmic domain. Finally, we generated two additional BarA-PD structural models, based on the sensor domains of DcuS and CitA and explored the relative position of the above-mentioned BarA residues. As expected, Leu95, Thr98, and Ile130 of BarA overlap with residues Arg107, His110, and Arg147 of DcuS, and Arg109, His112, and Arg150 of CitA that are needed for ligand binding in these two proteins (Fig. S2). It is relevant to mention that the discrepancy in identified amino acid residues, important for ligand binding in BarA between the amino acid alignment (Fig. 2*B*) and the structure-based alignment (Fig. S2) highlights the limitation of approaches based on the sequence alignments for the analysis of poorly conserved domains. Indeed, the BarA-PD shares 19%, 22%, and 18% identity with the Cache domains of GacS, DcuS, and CitA at the amino acid sequence level, respectively. Taken together, our results indicate that the Cache folded periplasmic region of BarA is a functional detector domain and implicate residues Leu95, Thr98, and Ile136 to be directly involved in signal binding. Curiously, these amino acid residues are not basic, as would be expected for a positively charged pocket able to bind a ligand bearing an anionic carboxylate, such as acetate or formate, but instead have polar hydroxyl group or hydrophobic side chains, leading us to reconsider the nature of the physiological stimulus of BarA.

### The protonated (neutral) state of formic and acetic acids provides the stimulus for the BarA/UvrY TCS

**Figure 4:**
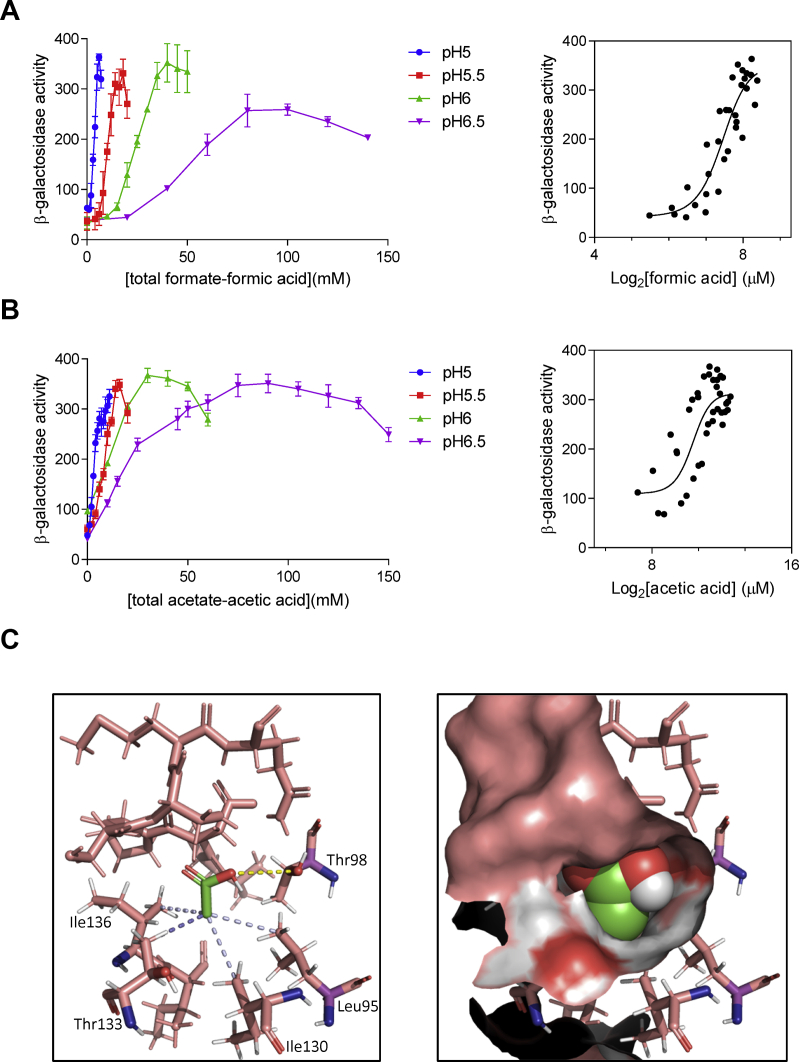
**The neutral state of formic and acetic acids provides the stimulus of BarA.***A*, the strain KSB837 (WT) was grown in LB medium buffered at pH 5.0, 5.5, 6.0, or 6.5 to an A_600_ of 0.15, and a designated amount of formate was added to the culture medium (0, 1, 2, 3, 4, 5, 6, or 7 mM for the culture at pH 5.0; 0, 4, 6, 8, 10, 12, 14, 16, 18, or 20 mM for the culture at pH 5.5; 0, 10, 15, 20, 25, 30, 35, 40, 45, or 50 mM for the culture at pH 6.0; and 0, 20, 40, 60, 80, 100, 120, or 140 mM for the culture at pH 6.5). The cultures were incubated for 60 min before the samples were withdrawn for β-galactosidase quantification. *B*, the strain KSB-ackA-pta was grown in LB medium buffered at pH 5.0, 5.5, 6.0, or 6.5 to an A_600_ of 0.15, and a designated amount of acetate was added to the culture medium (0, 1, 2, 3, 4, 5, 6, 7, 8, 9, 10, or 11 mM for the cultures at pH 5.0; 0, 2, 4, 6, 8, 10, 12, 14, 16, or 20 mM for the culture at pH 5.5; 0, 10, 20, 30, 40, 50, or 60 mM for the culture at pH 6.0; and 0, 10, 15, 25, 30, 45, 50, 60, 75, 90, 105, 120, 135, or 150 mM for the culture at pH 6.0). The cultures were incubated for 60 min before the samples were withdrawn for β-galactosidase quantification. *Left panels*, the β-galactosidase activity is plotted against the total concentration of formate – formic acid (*A*) or acetate – acetic acid (*B*) in the cultures grown at pH 5.0 (*blue*), pH 5.5 (*red*), pH 6.0 (*green*), and pH 6.5 (*purple*). The average from three independent experiments is presented, and the SDs (*error bars*) are indicated (*Right panels*). The nonlinear regression-fitting curve of the β-galactosidase activity plotted against the binary logarithm of the concentration of formic acid (*A*) or acetic acid (*B*). For clarity, only the average from the three independent experiments is presented in these panels. *C*, (*left panel*) position and atomic interaction of the acetic acid molecule into the binding site of the BarA sensor domain, as predicted by cavity detection and molecular docking. The acetic acid molecule is shown in a *stick representation* with carbon atoms colored *green* and oxygen atoms colored *red*. Only the BarA residues comprising the acetic acid-binding cavity are shown. The *dashed lines* represent the predicted hydrogen bond (*yellow*) and the hydrophobic interactions (*light blue*) between the acetic acid molecule and amino acid residues Leu95, Thr98, Ile130, Thr133, and Ile136 of BarA. *Right panel*, occupancy of the cavity by the ligand at the predicted BarA-binding pocket. The cavity of the BarA-PD is illustrated in a *surface style*, and the acetic acid molecule is shown in a *spheres representation*.

We previously demonstrated that the end products formate and acetate provide a physiological stimulus for BarA, and that an accessible carboxylate group in the signal molecule is essential for BarA activation (15). Now, we found that the putative ligand-binding pocket in the sensor domain of BarA seems to be comprised of hydrophobic and hydroxylated residues, rather than positively charged residues. This fact prompted us to speculate that the protonated state of acetate, acetic acid, or of formate, formic acid, may provide the physiological stimuli for the BarA-sensor kinase. To explore this possibility, the effect of increasing concentrations of formate to growth media buffered at different pHs on the activity of BarA/UvrY was examined. The following conditions were tested: LB medium buffered at (i) pH 5.0 in the presence of 0, 1, 2, 3, 4, 5, 6, or 7 mM formate, (ii) pH 5.5 in the presence of 0, 4, 6, 8, 10, 12, 14, 16, 18, or 20 mM formate, (iii) pH 6.0 in the presence of 0, 10, 15, 20, 25, 30, 35, 40, 45, or 50 mM formate, and (iv) pH 6.5 in the presence of 0, 20, 40, 60, 80, 100, 120, or 140 mM formate. In all cases, the cells were grown to an optical density at A_600_ of 0.15 before the indicated amount of formate was added to the culture medium, and the samples were withdrawn after 60 min for β-galactosidase activity determination. It was observed that the higher the pH, the greater amount of formate was needed to activate *csrB-lacZ* reporter expression (Fig. 4*A*). Moreover, when the obtained β-galactosidase activities were plotted against the log_2_ of the formic acid concentration, calculated by using the Henderson–Hasselbalch equation and pKa values of formic acid/formate of 3.75, a correlation between the *csrB-lacZ* expression and the formic acid concentration was observed (Fig. 4*A*), revealing a threshold concentration of 6.5 to 7 μM formic acid to be required for BarA activation, regardless of the pH. To provide further support to this conclusion, we carried out a similar experiment but now adding acetate to the growth media. In this case, to prevent acetyl-P-dependent UvrY phosphorylation, a strain in which the acetyl-P synthetic pathways were blocked by deletion of the acetate kinase (*ackA*) and phosphotransacetylase (*pta*) genes was used. The *ackA*::Tet^r^::*pta* mutant strain, carrying the *csrB-lacZ* reporter, was grown in LB medium buffered at pH 5.0, 5.5, 6.0, or 6.5, to an absorbance at A_600_ of 0.15. Thereafter, the indicated amount of acetate was added to the growth medium, and the cultures were incubated for 60 min before the samples were withdrawn for β-galactosidase quantification (Fig. 4*B*). It was observed that a higher concentration of acetate was needed to activate reporter expression at higher pH (Fig. 4*B*), and the β-galactosidase activity values correlated with the calculated acetic acid concentration (pKa_acetic acid/acetate_: 4.75), providing further support to the conclusion that the protonated state of acetate or formate provide the physiological stimulus of BarA. This is consistent with the occurrence of a noncharged-binding pocket in the sensor domain of BarA. To strengthen our conclusion, we performed an acetic acid binding site prediction on the BarA-PD model, by molecular docking enhanced by cavity detection (39, 40). The acetic acid molecule was docked into a cavity, which comprised the residues Leu95, Thr98, Leu103, Asp104, Pro105, Ser106, Ser107, Met108, Gln109, Ile130, Thr133, Ile135, and Ile136 (Fig. 4*C*), where the hydroxyl group of Thr98 associates with the protonated carboxylic group of acetic acid through hydrogen bonding, and the hydrophobic side chains of Leu95, Ile130, Thr133, and Ile136 are predicted to interact with the carbon chain of the organic acid (Fig. 4*C*). Taken together, our results suggest that a functional region within the BarA PD, which consists of a noncharged binding pocket, is involved in acetic acid or formic acid binding.

### Differential evolution of the BarA orthologs

**Figure 5:**
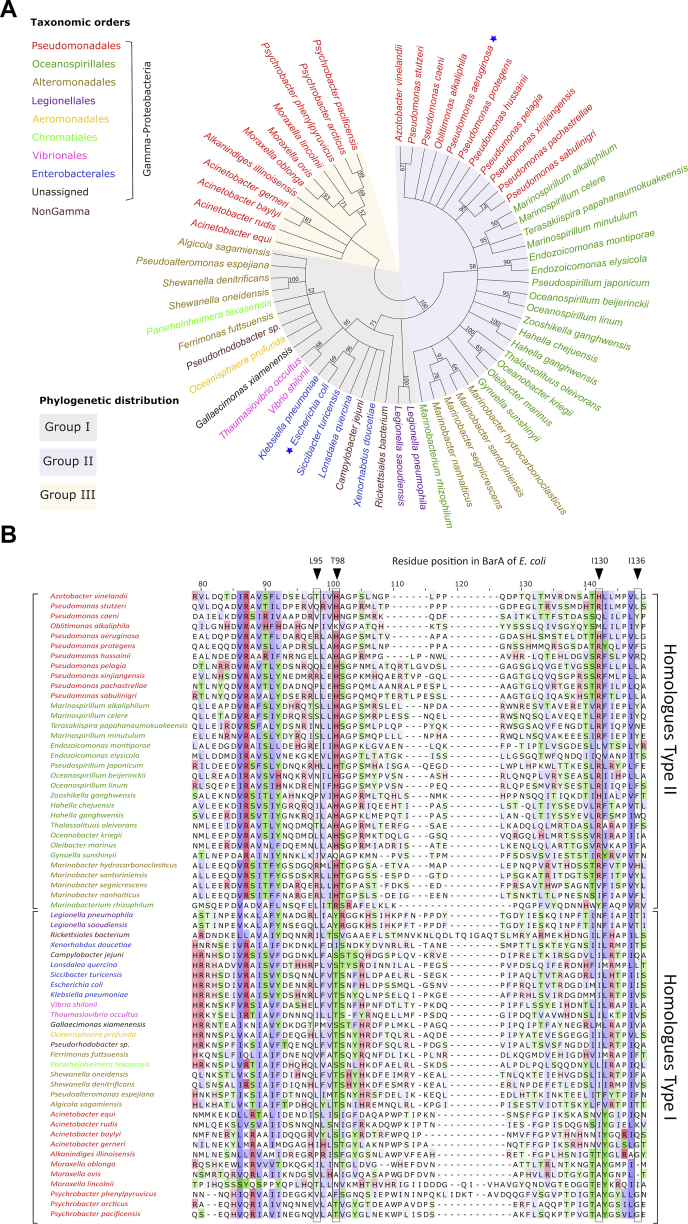
**Sequence analysis and phylogenetic distribution of the putative sensor domain of BarA orthologous proteins.***A*, phylogenetic tree based on the amino acid sequences of the periplasmic domains of BarA homologs. The tree was arbitrarily rooted and condensed so that only bootstrap support values higher than 50% are shown. The branches were grouped and shaded different colors according to the phylogenetic distribution. The taxon names of bacterial species are color coded according to the taxonomic order designation. The position of BarA of *Escherichia coli* and GacS of *Pseudomonas aeruginosa* in the tree is marked with a *blue star*. *B*, multiple sequence alignment of the 63 nonredundant periplasmic domains of BarA orthologs. For clarity, only the central portion of the alignment is shown. The *boxes* indicate conserved residues that are predicted to be involved in signal binding, and their position in the sequence of BarA of *E. coli* is shown. The brackets enclose bacterial taxa and sequences grouped according to the nature of these residues (see text).

The BarA/UvrY TCS of *E. coli* exerts global regulatory effects on gene expression by activating CsrB and CsrC transcription, thereby controlling CsrA activity (2, 9, 41). This signaling circuitry is highly conserved in γ-proteobacteria (42, 43, 44), preserving most of its general characteristics. However, the genes whose expression are modulated by the BarA/UvrY/CsrA regulatory cascade and hence the physiological response, may vary significantly among bacteria (10, 45, 46, 47, 48, 49, 50, 51). Here, we found that the input domain of the BarA/UvrY system of *E. coli* differs from that of the homologous GacS/GacA of *P. aeruginosa,* in that the former has evolved to recognize an uncharged signal. To gain further insight into the evolution and the diversity of the putative sensor domains of BarA orthologous proteins, we examined the phylogenetic relationships of the PDs in a group of species selected from fully sequenced bacteria encoding an identifiable BarA ortholog. About 500 deduced protein sequences were acquired from the GenBank database, using the Basic Local Alignment Search Tool (52), and the sequence of the BarA and GacS periplasmic domains as query. An unrooted maximum-likelihood phylogenetic tree was inferred from the nonredundant sequences (identity lower than 50%), which included members of nine gamma, two alpha, and one epsilon proteobacterial orders. The phylogenetic reconstruction showed that BarA orthologues could be classified into three main groups. Firstly, the bacteria belonging to the order Enterobacteriales cluster with the orders Vibrionales, Aeromonadales, Chromatiales, Legionellales, and various families from the order Alteromonadales in addition to the members of alpha and epsilon proteobacteria (group I) (Fig. 5*A*). Secondly, the BarA/GacS sensor domain seems to have early diverged within Pseudomonadales, because bacteria within the family Pseudomonadaceae were clustered together with members of the orders Oceanospirillales and Alteromonadales (group II) (Fig. 5*A*). Finally, genera belonging to the family Moraxellaceae was shown to be gathered in a separated branch (group III) (Fig. 5*A*). Moreover, a multiple-sequence alignment of the 63 nonredundant BarA-PD sequences revealed important differences in the putative signal-binding pocket of the three afore-mentioned phylogenetic groups. Particularly, most of the BarA homologs clustered into group II, represented by GacS of *P. aeruginosa*, are characterized by a conserved residue pattern that contains the positively charged residues His and/or Arg at two specific positions (Fig. 5*B*). This pair of highly conserved residues (His97 and His133) has been shown to be essential for GacS/GacA signaling in *P. aeruginosa* (17). In contrast, members of group I, represented by the BarA of *E. coli*, contain highly conserved Ser/Thr/Tyr residues at the first position (Thr98 in BarA of *E. coli*) and Ile/Leu/Val residues at the second position (Ile130 in BarA of *E. coli*), (Fig. 5*B*). It is worth mentioning that although the replacement of Ile130 by Ala did not have an effect on the BarA function, this residue could be involved in signal detection (Figs. 4*C* and S2), because it is highly conserved in the BarA homologs Type I and because alanine is also a hydrophobic side-chain residue. Finally, the amino acid pattern of the putative-binding pocket of the BarA proteins clustered in group III is closer related to that of Type I because most of them contain conserved hydroxylated and hydrophobic residues in the two positions, suggesting that the detector domain of these HKs could also interact with noncharged signals. Thus, the differential residue conservation pattern of the putative signal-binding pocket, taken together with our genetic analysis for the BarA-PD and with the previously reported characterization of the GacS-PD (17), strongly suggest that Type I and Type II BarA proteins diverged and evolved separately to detect different kind of signal molecules.

## Conclusions

We previously showed that the end products formate and acetate constitute the physiological signal for the activation of the BarA/UvrY TCS of *E. coli*. In this work, we explored the role of the periplasmic region of the BarA HK in stimulus perception. Our results demonstrate that the PD of BarA constitutes a functional domain that is required for activation of the BarA–UvrY signaling pathway in response to the physiological stimulus. Our functional analyses combined with protein structure prediction and molecular docking indicate that the putative ligand-binding pocket of the BarA PD does not depend on positively charged residues, as would be expected for the stabilization of a negatively charged carboxyl group. Instead, it was found that the ligand-binding pocket of the BarA PD depends on polar hydroxyl groups or hydrophobic side chains, and that the neutral state of the carboxyl group moiety of acetic or formic acid provides the stimulus for the BarA sensor kinase, providing an explanation for the above observation. Nevertheless, the conclusion that acetic acid and formic acid directly activate BarA remains to be further supported by biochemical analysis with the purified sensor domain. Thus far, we have been unsuccessful in demonstrating the binding of acetic or formic acid to the purified periplasmic domain of BarA. One possible explanation could be the fact that the purified protein was shown to be insoluble at pHs below 7.0 (data not shown), frustrating the binding experiments at pHs at which the concentration of the protonated carboxylic acid were high enough to observe the interaction. However, the immediate response to the stimuli and the fact that acetic and formic acid are not readily converted to any common intermediary metabolite strongly suggest that their related chemical structures permit them to signal directly to BarA. Finally, our phylogenetic analysis prompted us to propose that the two families of BarA orthologous proteins have evolved separately to acquire differential signal-binding properties by their sensor domains, permitting these HKs to perceive different kinds of signal molecules, thereby highlighting the plasticity of this family of signaling proteins.

## Experimental procedures

### Bacterial strains, plasmids, and growth conditions

The *E. coli* strains and plasmids used in this work are listed in Table S1. Strain IFC5035 (Δ*barA*::Kan^r^ *csrB-lacZ*) was constructed by homologous recombination using the lambda-red recombinase system (53). Briefly, a PCR-amplified fragment, using primers barAdel-Fw and barAdel-Rv (Table S2) and plasmid pKD4 (53) as the template, was used to replace the *barA* gene with a kanamycin (Kan) cassette in strain KSB837 (WT, *csrB-lacZ*) (7). Then, the FRT-flanked Kan^r^ cassette was removed from the strain IFC5035 using the Flp recombinase encoded in the temperature-sensitive plasmid pCP20 (54), obtaining the strain IFC5036 (Δ*barA csrB-lacZ*).

To construct plasmid pUC18-barA, the *barA* coding sequence was amplified by PCR using the primers barA-NdeI-Fw and BAfullR-Hind (Table S2), and the chromosomal DNA of strain KSB837 as a template. The PCR product was digested with NdeI-HindIII and cloned between the NdeI-HindIII sites of plasmid pUC18 (55), resulting in the plasmid pUC18-barA. To construct the low-copy number plasmid pEXT22-barA, which carries the *barA* native promoter and ribosomal binding site, an introduced NdeI site that includes the initiation codon of *barA* and the *barA* open reading frame, a 122-bp DNA fragment containing the transcriptional regulatory region of the *barA* gene was PCR-amplified using the primers BarA100up-Fw and BA5R (Table S2) and the chromosomal DNA of strain KSB837 as a template. The PCR product was digested with MluI-XbaI and cloned into the MluI-XbaI sites of a modified pEXT22 vector (56) (the NdeI site of pEXT22 was destroyed by cleaving, filling in, and ligation). Subsequently, the *barA* open reading frame was isolated as a 2.8 Kb NdeI-HindIII fragment from the plasmid pUC18-barA and inserted into the NdeI and HindIII sites of the above construct, resulting in the plasmid pEXT22-barA.

Bacteria were routinely cultured at 37 °C in LB medium. The LB agar medium was prepared by the addition of 1.5% (w/v) agar. When required, the LB medium was buffered at pH 7.0 with 100 mM 3-(N-morpholino)propanesulfonic acid; at pH 6.5, pH 6.0, and pH 5.5 with 100 mM 2-(N-morpholino)ethanesulfonic acid; and at pH 5.0 with 100 mM homopiperazine-1,4-bis(2-ethanesulfonic acid). The used concentration of the buffers was sufficient to maintain a constant pH throughout all the experiments. When necessary, the growth medium was supplemented with chloramphenicol (20 μg/ml), kanamycin (50 μg/ml), ampicillin (100 μg/ml), or tetracycline (10 μg/ml). For semiquantitative detection of the *csrB-lacZ* reporter expression, LB agar was supplemented with 5-bromo-4-chloro-3-indolyl-β-D-galactoside (X-gal) (40 μg/ml). When indicated, acetate and formate were added to the growth media at a concentration of 7 mM, unless otherwise stated.

### Generation of plasmids expressing cytosolic or chimeric BarA proteins

To generate a low-copy number plasmid expressing the cytosolic portion of the BarA protein (BarA^Δ1–197^), a PCR amplified DNA fragment, using the primers BarA5′(198-x) and BAfullR-Hind (Table S2) and chromosomal DNA of strain KSB837 as a template, was used to replace the NdeI-HindIII restriction fragment of pEXT22-barA to generate pEXT22-barAcyt. A *barA*-*gacS* fusion, encoding a hybrid protein in which the periplasmic segment of BarA was replaced by the corresponding section of GacS of *A. vinelandii*, was constructed by a three-step PCR procedure (57). Firstly, a 453-bp DNA fragment was PCR-amplified using the primers ChGS-perip-Fw and ChGS-perip-Rv (Table S2) and chromosomal DNA of *A. vinelandii* as a template. Secondly, the purified PCR product was used in combination with plasmid pEXT22-barA as templates in two PCR reactions, one of which using the primers GS-perip-Rv and BarA100up-Fw, and the other one containing primers GS-perip-Fw and BAfullR-Hind (Table S2), to amplify a 559-bp DNA and a 2664-bp DNA product, respectively. Finally, the full-length barA-gacS fusion was PCR-amplified by using the primers BarA100up-Fw and BAfullR-Hind and the purified PCR products from the previous step as templates. The resulting 2867-bp DNA product was digested with NdeI and HindIII, and the 2.7 Kb fragment was gel-purified and used to replace the NdeI-HindIII restriction fragment of pEXT22-barA to generate pEXT22-barAPDgacS. To construct the *barA*-*arcB* fusion, encoding a hybrid protein in which the periplasmic segment of BarA was replaced by the short periplasmic bridge of ArcB, a PCR reaction was performed using primers Arc-Bar-peripl-Fw and Arc-Bar-peripl-Rv (Table S2) and pEXT22-barA as a template. The 7508-bp length PCR product was purified and circularized by self-ligation to generate pEXT22-barAPDarcB.

### Generation of chromosomal barA mutants by site-directed mutagenesis

To generate KSB837 derivative strains harboring chromosomal *barA* mutant alleles, a series of plasmids were created as follows. Plasmid pUC18-Cam was constructed by cloning a 1033-bp PCR-amplified fragment containing an FRT-flanked chloramphenicol resistance (*cat*) gene [using pKD-Kpn-Hind-Fw and pKDEco2-Rv (Table S2) as primers and plasmid pKD3 (53) as a template] into the HindIII-EcoRI sites of pUC18. BarA punctual mutant variants (*barA*∗) were created by site-directed mutagenesis according to a two-step PCR procedure (58). The first PCR amplifications were performed using the plasmid pUC18-barA as a template and barA-NdeI-Fw and either BarA-S99A-Rv, BarA-H102A-Rv, BarA-R124A-Rv, BarA-I130A-Rv, or BarA-R132A-Rv as primers (Table S2). Each purified product was used as a primer with primer BAfullR-Hind for the second PCR, using the plasmid pUC18-barA as a template. The second PCR products were digested with NdeI and HindIII and cloned between the corresponding sites of vector pUC18-Cam, resulting in pBarAS99ACam, pBarAH102ACam, pBarAR124ACam, pBarAI130ACam, and pBarAR132ACam. To construct the strains IFC5038 (*barA*^S99A^::Cam^r^), IFC5039 (*barA*^H102A^::Cam^r^), IFC5040 (*barA*^R124A^-Cam^r^), IFC5041 (*barA*^I130A^-Cam^r^), and IFC5042 (*barA*^R132A^-Cam^r^), PCR-amplified fragments, using primers the barAmut-ins-Fw and barAdel-Rv (Table S2), and plasmids pBarAS99ACam, pBarAH102ACam, pBarAR124ACam, pBarAI130ACam, or pBarAR132ACam as the template, were used to replace the Δ*barA* allele in the strain IFC5036 with the corresponding mutant *barA*∗::Cam^r^ allele by homologous recombination using the lambda-red recombinase system (53).

### *In vitro* random mutagenesis of barA

To construct the plasmid pDuetBarAXho, which was used as a template for error-prone PCR, the *barA* open reading frame was PCR-amplified, using the primers barAf1NcoI and BAfullR-Hind (Table S2) and chromosomal DNA of strain KSB837 as the template, and cloned between the NcoI-HindIII sites of a modified pACYCDuet-1 (Novagen) [pACYCDuet-1(-XhoI), in which the XhoI site was destroyed by cleaving, filling in, and ligation], resulting in pDuetBarA. A silent mutation that created an XhoI restriction site in *barA*, at a position corresponding to the second transmembrane domain of BarA, was introduced by site-directed mutagenesis of plasmid pDuetBarA using the QuikChange kit (Stratagene) and the mutagenic primers BarAXhoITM2-Fw and BarAXhoITM2-Rv (Table S2), resulting in the pDuetBarAXho plasmid. Random mutagenesis of the N-terminal region of BarA was performed by error-prone PCR according to the method of Cadwell and Joyce (59). The reaction mixtures contained 5 ng of plasmid pDuetBarAXho as a template, 2 μM of primers barAf1NcoI and BarAXhoITM2-Rv, 50 mM KCl, 10 mM Tris-HCl buffer (pH 8.3), 7 mM MgCl_2_, 0.5 mM MnCl2, 0.2 mM each dATP and dGTP, 1 mM each dTTP and dCTP, and 5 U of Taq DNA polymerase (New England Biolabs) in a total volume of 100 μl. The PCRs were performed with 20 cycles of the following steps: (i) 95 °C for 30 s, (ii) 60 °C for 30 s, and (iii) 72 °C for 1 min. To generate a mutant library, the purified PCR products were used to replace the NcoI-HindIII restriction fragment of pDuetBarAXho. The resulting library was introduced into the strain IFC5035 (Δ*barA*::Kan^r^ *csrB-lacZ*) by transformation, and the individual clones were screened by streaking on suitable agar plates, as described above.

### Determination of β-galactosidase activity

The cells of KSB837 derivative strains, carrying the UvrY-P–activatable *csrB-lacZ* reporter, were grown aerobically in LB adjusted to and buffered at the indicated pH, at 37 °C. When indicated, acetate or formate were added to the growth media. The samples were withdrawn at the indicated times or A_600_, and β-Galactosidase activity was assayed and expressed in Miller units, as described previously (60).

### Subcellular fractionation and Western blot analysis

The cultures grown aerobically in LB were harvested during midexponential growth, and the cells were washed with Tris/HCl buffer (50 mM Tris/HCl pH 7.6, 200 mM KCl, 10 mM MgCl2, and 0.1 mM EDTA). The cell pellet was resuspended in 5 ml of the same buffer and disrupted by sonication. Cell debris was removed by centrifugation for 10 min at 10,000*g*. The supernatant was centrifuged for 45 min at 30,000*g* to separate the cytosolic and the membrane fractions. The resultant supernatant fluid containing the soluble proteins was collected. The remaining pellet was resuspended in 0.5 ml of Tris/HCl buffer. The samples of cytosolic and membrane (containing 5 μg of protein) fractions were separated by SDS-PAGE (10% polyacryl-amide gel), and the proteins were transferred to a Hybond-ECL filter (Amersham Biosciences). The filter was equilibrated in TTBS buffer (25 mM Tris, 150 mM NaCl, and 0.05% Tween 20) for 10 min and incubated in blocking buffer (1% milk in TTBS) for 1 h at room temperature. BarA polyclonal antibodies, raised against His6-BarA^198–918^ (33), were added at a dilution of 1:10,000 and incubated for 1 h at room temperature. The bound antibody was detected by using anti-rabbit IgG antibody conjugated to horseradish peroxidase and the Immobilon Western detection system (Millipore). The protein bands were quantified using ImageJ software (61).

### Structural modeling and binding site prediction

The Iterative Threading Assembly Refinement server (37, 62) was used to model the BarA PD (residues 31–178), using the GacS periplasmic domain (5O7J) as a template, which shares 19% identity and 44% similarity at the protein level. The generated model had a C-score of 1.12 and a TM-score of 0.73 ± 0.11. The model was improved by full-atomic simulations using ModRefiner (63). The final model has 76.7% of all residues residing in the most-favored region of the Ramachandran plot and 19.5% of residues in additionally allowed regions, as calculated by the PROCHECK module of the PDBSum server (64). The acetic acid (in its protonated form) binding site of BarA-PD was predicted by molecular docking simulations, using the CB-Dock server (39), which detects protein cavities to guide blind docking by the algorithm AutoDock Vina (40). The ribbon structures were viewed and aligned, and the figures were rendered using PyMOL (The PyMOL Molecular Graphics System, version 2.0, Schrödinger).

### Phylogenetic analysis

Approximately, 500 protein sequences were collected from the GenBank nonredundant protein sequences database by a BLASTP search using the BarA or the GacS periplasmic domain sequence as a seed. The redundant sequences and those with identities higher than 50% were excluded, resulting in a dataset of 63 protein sequences (NCBI accessions are shown in Table S3). Multiple-sequences alignments were performed by MUSCLE (65). The phylogenetic and molecular evolutionary analyses were conducted by the Maximum Likelihood method using MEGA version X (66). The best-fit models of amino acid substitution were selected by the Bayesian information criterion. The bootstrap confidence levels were obtained using 500 replicates. Phylogenetic tree was visualized and arbitrarily rooted using the midpoint rooting method implemented in Mega software (66), and the tree was condensed so that only bootstrap support values higher than 50% are shown.

### Statistics

All the quantitative experiments were performed in triplicates, and the experimental results are expressed as mean ± the SD value.

## Data availability

All data are contained in the article and in the Supporting information.

## Supporting information

This article contains supporting information (7, 15, 53, 55, 56).

## Conflict of interest

The authors declare that they have no conflicts of interest with the contents of this article.
