# Supplementary material for: The Escherichia coli two-component signal sensor BarA binds protonated acetate via a conserved hydrophobic-binding pocket
Source: J Biol Chem. 2021 Nov 4;297(6):101383. doi: 10.1016/j.jbc.2021.101383 (PMC8639471; doi:10.1016/j.jbc.2021.101383)
Supplement: Figures S1 and S2 and Tables S1–S3 [file mmc1.pdf]

Supplementary Information for

The *E. coli* two-component signal sensor BarA binds protonated acetate via a conserved hydrophobic binding pocket

Adrián F. Alvarez, Claudia Rodríguez, Ricardo González-Chávez, Dimitris Georgellis*

*Dimitris Georgellis
Email: dimitris@ifc.unam.mx

This PDF file includes:

Figure S1

Figure S2

Table S1

Table S2

Table S3

Figure S1

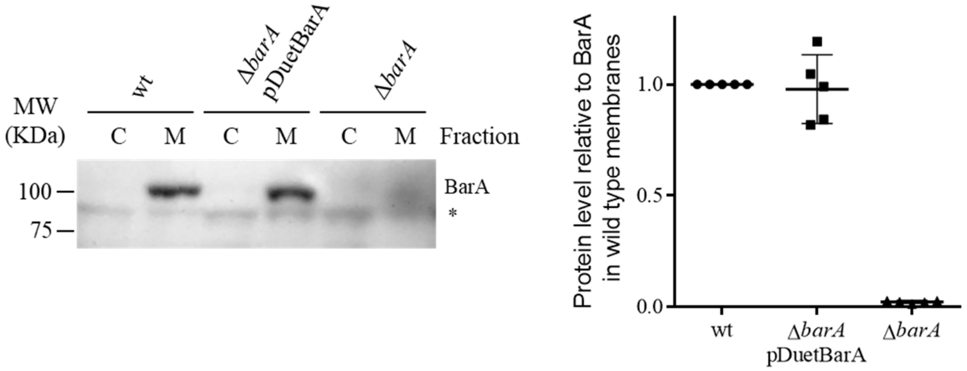

Fig. S1. **Complementation of a *barA* mutant strain with plasmid pDuetBarA results in nearly wild-type BarA levels.** BarA protein (102.5 KDa) levels in cytosolic (C) and membrane (M) fractions of strains KSB837 (WT), IFC5035 (*barA*$^{-}$) carrying the pDuetBarA plasmid, and IFC5035 (*barA*$^{-}$), as determined by Western blot analyses using BarA polyclonal antibodies. A quantification from five independent blots is shown on the right, indicating mean ± SD, with individual values indicated (wt, filled circles; IFC5035 (Δ*barA*) carrying pDuetBarA, filled squares; and IFC5035 (Δ*barA*), triangles). * indicates a nonspecific band present in all stains and fractions.

Figure S2

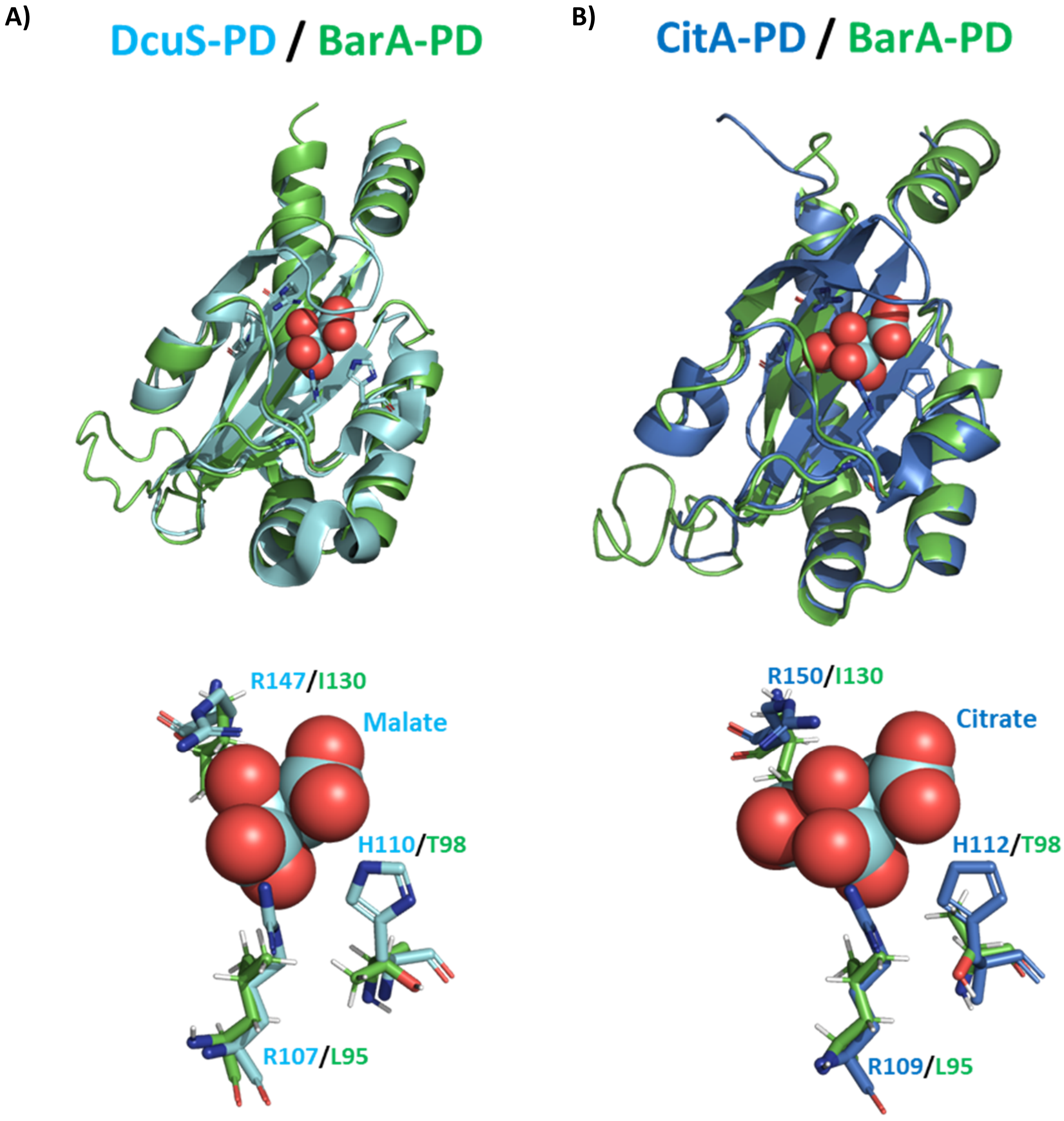

Fig. S2. Structural models of BarA-PD based on the DcuS and CitA sensor domains. (A) (Upper panel) Cartoon of the structural model of BarA-PD generated by using the program I-Tasser and the crystal structure of the DcuS sensor domain as the template (PDB: 3BY8). The BarA-PD model (green) and the template (light blue), which includes the bound malate molecule, are shown overlapped. (Lower panel) The relative positions of the DcuS amino acid residues needed for malate binding (light blue) to the corresponding BarA amino acid residues (green), in the generated model, are depicted. (B) (Upper panel) Cartoon of the structural model of BarA-PD generated by using the program I-Tasser and the crystal structure of the CitA sensor domain as the template (PDB: 1P0Z). The BarA-PD model (green) and the template (blue), which includes a bound citrate molecule, are shown overlapped. (Lower panel) The relative positions of the CitA amino acid residues needed for citrate binding (light blue) to the corresponding BarA amino acid residues (green), in the generated model, are depicted.

Table S1. Bacterial strains and plasmids used in this study

Strain or plasmid	Relevant characteristics	Source or reference
**Strain**		
KSB837	CF7789 λϕ(*csrB′-lacZ*)	(7)
IFC5035	KSB837 Δ*barA*::Kan^{r}	This work
IFC5036	KSB837 Δ*barA*	This work
IFC5038	KSB837 *barA*^{S99A}::Cam^{r}	This work
IFC5039	KSB837 *barA*^{H102A}::Cam^{r}	This work
IFC5040	KSB837 *barA*^{R124A}::Cam^{r}	This work
IFC5041	KSB837 *barA*^{I130A}::Cam^{r}	This work
IFC5042	KSB837 *barA*^{R132A}::Cam^{r}	This work
KSB-ackA-pta	KSB837 *ackA*::Tet^{r}::*pta*	(15)
**Plasmid**		
pEXT22	Low copy number vector, Kan^{r}	(56)
pEXT22(-NdeI)	pEXT22 derivative, carries 2-bp insertion as a result of end filling and ligation at the NdeI site	This work
pEXT22-barA	*barA* under native promoter in pEXT22(-NdeI)	This work
pEXT22-barAcyt	*barA*$^{198\text{-}918}$ under native promoter in pEXT22(-NdeI)	This work
pEXT22-barAPDarcB	*barA*$^{1\text{-}31}$-*arcB*$^{43\text{-}57}$-*barA*$^{176\text{-}918}$ under *barA* native promoter in pEXT22(-NdeI)	This work
pEXT22-barAPDgacS	*barA*$^{1\text{-}34}$-*gacS*$^{23\text{-}154}$-*barA*$^{176\text{-}918}$ under *barA* native promoter in pEXT22(-NdeI)	This work
pUC18	Cloning vector, Amp^{r}	(55)
pUC18-barA	*barA*^{WT} in pUC18	This work
pUC18-Cam	Cloning vector, Amp^{r} Cam^{r}	This work
pBarAS99ACam	*barA*^{S99A}::Cam^{r} in pUC18	This work
pBarAH102ACam	*barA*^{H102A}::Cam^{r} in pUC18	This work
pBarAR124ACam	*barA*^{R124A}::Cam^{r} in pUC18	This work
pBarAI130ACam	*barA*^{I130A}::Cam^{r} in pUC18	This work
pBarAR132ACam	*barA*^{R132A}::Cam^{r} in pUC18	This work
pKD3	Template plasmid for *cat* cassette amplification flanked by FRT sequences, Amp^{R}, Cam^{r}	(53)
pKD4	Template plasmid for *kan* cassette amplification flanked by FRT sequences, Amp^{R}, Kan^{r}	(53)
pACYCDuet-1	T7 promoter/*lac* operator expression vector, Cam^{r}	Novagen
pACYCDuet-1(-XhoI)	pACYCDuet-1 derivative, carries 4-bp insertion as a result of end filling and ligation at the XhoI site	This work
pDuetBarA	*barA* under the control of the T7 promoter in pACYCDuet-1(-XhoI)	This work
pDuetBarAXho	*barA* with an inserted XhoI restriction site by silent mutation (*barA*^{XhoI}) in pACYCDuet-1(-XhoI)	This work

pDuetBarAL52P	*barA*$^{XhoI\ L52P}$ in pACYCDuet-1(-XhoI)	This work
pDuetBarAV83D	*barA*$^{XhoI\ V83D}$ in pACYCDuet-1(-XhoI)	This work
pDuetBarAV88D	*barA*$^{XhoI\ V88D}$ in pACYCDuet-1(-XhoI)	This work
pDuetBarAL95F	*barA*$^{XhoI\ L95F}$ in pACYCDuet-1(-XhoI)	This work
pDuetBarAT98A	*barA*$^{XhoI\ T98A}$ in pACYCDuet-1(-XhoI)	This work
pDuetBarAT98H	*barA*$^{XhoI\ T98H}$ in pACYCDuet-1(-XhoI)	This work
pDuetBarAI136N	*barA*$^{XhoI\ I136N}$ in pACYCDuet-1(-XhoI)	This work

Table S2. Oligonucleotides used in this study

Primer name	Primer sequence (5´-3')	Use
BarA100up-Fw	GACGCGTCGTCGTGCGTC	Used to amplify the *barA* promoter region
BA5R	TGCTCTAGAAGATCTGATCATATGGAGTTCCGTTATGGG	Used to amplify the *barA* promoter region
barA-NdeI-Fw	CAACATATGACCAACTACAGCCTGCGC	Used to amplify the full-lenght *barA* gene and the megaprimers to generate *barA* punctual mutants
BAfullR-Hind	CCCAAGCTTGCCGATTGCTACTCGACAAGA	Used to amplify the full-lenght *barA* gene
BarA5'(198-x)	CCCGGATCCCATATGCGCGATGTAACCGGTCCG	Used to amplify the coding sequence of the cytosolic BarA variant
ChGS-perip-Fw	CTGAGTATCTTTTTCGTCGTGCATCGCTATTTGGCCGAGAT GCACGGC	Used to construct the $BarA^{PDGacS}$ chimeric HK
ChGS-perip-Rv	CATCATCACGCTGGAAATAAAGATCTCGCGGTAGCCCTGC AGCAG	Used to construct the $BarA^{PDGacS}$ chimeric HK
GS-perip-Rv	GCGGTAGCCCTGCAGCAG	Used to construct the $BarA^{PDGacS}$ chimeric HK
GS-perip-Fw	TTGGCCGAGATGCACGGC	Used to construct the $BarA^{PDGacS}$ chimeric HK
Arc-Bar-peripl-Fw	GTCGAAAGCATTGATGTTATTCGTGAGATCTTTATTTCCAG CGTG	Used to construct the $BarA^{PDArcB}$ chimeric HK
Arc-Bar-peripl-Rv	CTGACCATGCAGCACCATGGTTACGACGAAAAAGATACTC AGC	Used to construct the $BarA^{PDArcB}$ chimeric HK
barAdel-Fw	ATTTAACAGTGTGACCTTAATTGTCCCATAACGGAACTCCG TGTAGGCTGGAGCTGCTTC	Used to delete the *barA* gene
barAdel-Rv	CATAAACACAGGCACTTTGTCACCAATCTGAAACCAGCGTA TGAATATCCTCCTTAGTTCC	Used to delete the *barA* gene and to integrate *barA* variants into the chromosome
pKDEco2-Rv	CGGAATTCATGAATATCCTCCTTAGTTC	Used to amplify the *cat* gene from

		plasmid pKD3
pKD-Kpn-Hind-Fw	GGGGTACCAAGCTTGTGTAGGCTGGAGCTGCTTC	Used to amplify the *cat* gene from plasmid pKD3
barAmut-ins-Fw	ATTTAACAGTGTGACCTTAATTGTCCCATAACGGAACTCCATGACCAACTACAGCCTGC	Used to integrate *barA* variants into the chromosome
BarA-S99A-Rv	GATCAAGATGAAAATTGGCGGTGACAAAGAGTCGG	Used to generate *barA* punctual mutant by megaprimer amplification
BarA-H102A-Rv	CATTGATGAGGGATCAAGAGCAAAATTGGAGGTGACAAAGAG	Used to generate *barA* punctual mutant by megaprimer amplification
BarA-R124A-Rv	GATCATAATATCGCCGTCAGCAGTGACAGTGAGCTGGCG	Used to generate *barA* punctual mutant by megaprimer amplification
BarA-I130A-Rv	GAAATAATCGGCGTGCGGAGGGCCATAATATCGCCGTCACG	Used to generate *barA* punctual mutant by megaprimer amplification
BarA-R132A-Rv	CTCAGAAATAATCGGCGTGGCGAGGATCATAATATCGCC	Used to generate *barA* punctual mutant by megaprimer amplification
barAf1NcoI	CAACCATGGCGACCAACTACAGCCTGCGCGCAC	Used to amplify the full-lenght *barA* gene and the *barA* periplasmic region
BarAXhoITM2-Fw	GCAGCAATATAAAGAGATCTTTATCTCGAGCGTGATGATGCTG	Used to create a XhoI restriction site in the BarA sequence by a silent mutation
BarAXhoITM2-Rv	CAGCATCATCACGCTCGAGATAAAGATCTCTTTATATTGCTGC	Used to create a XhoI restrcition site in the BarA sequence by a silent mutation and to ampify the *barA* periplasmic region

Table S3. NCBI reference sequence accessions of BarA orthologs used in phylogenetic analysis.

Accession	Organism	Class	Order	Family
**WP_197471059.1**	*Azotobacter vinelandii*	Gammaproteobacteria	Pseudomonadales	*Pseudomonadaceae*
**WP_186415863.1**	*Pseudomonas stutzeri*	Gammaproteobacteria	Pseudomonadales	*Pseudomonadaceae*
**WP_022965696.1**	*Pseudomonas caeni*	Gammaproteobacteria	Pseudomonadales	*Pseudomonadaceae*
**WP_053111169.1**	*Oblitimonas alkaliphila*	Gammaproteobacteria	Pseudomonadales	*Pseudomonadaceae*
**WP_034024066.1**	*Pseudomonas aeruginosa*	Gammaproteobacteria	Pseudomonadales	*Pseudomonadaceae*
**WP_011062714.1**	*Pseudomonas protegens*	Gammaproteobacteria	Pseudomonadales	*Pseudomonadaceae*
**WP_071871354.1**	*Pseudomonas hussainii*	Gammaproteobacteria	Pseudomonadales	*Pseudomonadaceae*
**WP_022960986.1**	*Pseudomonas pelagia*	Gammaproteobacteria	Pseudomonadales	*Pseudomonadaceae*
**WP_093397665.1**	*Pseudomonas xinjiangensis*	Gammaproteobacteria	Pseudomonadales	*Pseudomonadaceae*
**WP_083727290.1**	*Pseudomonas pachastrellae*	Gammaproteobacteria	Pseudomonadales	*Pseudomonadaceae*
**WP_092288012.1**	*Pseudomonas sabulinigri*	Gammaproteobacteria	Pseudomonadales	*Pseudomonadaceae*
**WP_072324365.1**	*Marinospirillum alkaliphilum*	Gammaproteobacteria	Oceanospirillales	*Oceanospirillaceae*
**WP_091961386.1**	*Marinospirillum celere*	Gammaproteobacteria	Oceanospirillales	*Oceanospirillaceae*
**WP_068997271.1**	*Terasakiispira papahanaumokuakeensis*	Gammaproteobacteria	Oceanospirillales	*incertae sedis*
**WP_027849455.1**	*Marinospirillum minutulum*	Gammaproteobacteria	Oceanospirillales	*Oceanospirillaceae*
**WP_051789246.1**	*Endozoicomonas montiporae*	Gammaproteobacteria	Oceanospirillales	*Endozoicomonadaceae*
**WP_020583285.1**	*Endozoicomonas elysicola*	Gammaproteobacteria	Oceanospirillales	*Endozoicomonadaceae*
**WP_177166874.1**	*Pseudospirillum japonicum*	Gammaproteobacteria	Oceanospirillales	*Oceanospirillaceae*
**WP_036567047.1**	*Oceanospirillum beijerinckii*	Gammaproteobacteria	Oceanospirillales	*Oceanospirillaceae*
**WP_077242806.1**	*Oceanospirillum linum*	Gammaproteobacteria	Oceanospirillales	*Oceanospirillaceae*
**WP_027706946.1**	*Zooshikella ganghwensis*	Gammaproteobacteria	Oceanospirillales	*Hahellaceae*
**WP_011395717.1**	*Hahella chejuensis*	Gammaproteobacteria	Oceanospirillales	*Hahellaceae*
**WP_020406163.1**	*Hahella ganghwensis*	Gammaproteobacteria	Oceanospirillales	*Hahellaceae*
**WP_015486099.1**	*Thalassolituus oleivorans*	Gammaproteobacteria	Oceanospirillales	*Oceanospirillaceae*
**WP_156879095.1**	*Oceanobacter kriegii*	Gammaproteobacteria	Oceanospirillales	*Oceanospirillaceae*
**TPD55691.1**	*Oleibacter marinus*	Gammaproteobacteria	Oceanospirillales	*Oceanospirillaceae*
**WP_044617057.1**	*Gynuella sunshinyii*	Gammaproteobacteria	Oceanospirillales	*Saccharospirillaceae*

**WP_041655427.1**	*Marinobacter hydrocarbonoclasticus*	Gammaproteobacteria	Alteromonadales	*Alteromonadaceae*
**WP_008937433.1**	*Marinobacter santoriniensis*	Gammaproteobacteria	Alteromonadales	*Alteromonadaceae*
**WP_091852392.1**	*Marinobacter segnicrescens*	Gammaproteobacteria	Alteromonadales	*Alteromonadaceae*
**WP_004583184.1**	*Marinobacter nanhaiticus*	Gammaproteobacteria	Alteromonadales	*Alteromonadaceae*
**WP_020680514.1**	*Marinobacterium rhizophilum*	Gammaproteobacteria	Oceanospirillales	*Oceanospirillaceae*
**WP_010947629.1**	*Legionella pneumophila*	Gammaproteobacteria	Legionellales	*Legionellaceae*
**WP_058535550.1**	*Legionella saoudiensis*	Gammaproteobacteria	Legionellales	*Legionellaceae*
**MBV35913.1**	*Rickettsiales bacterium*	Alphaproteobacteria	Rickettsiales	*unclassified Rickettsiales*
**WP_045972244.1**	*Xenorhabdus doucetiae*	Gammaproteobacteria	Enterobacterales	*Morganellaceae*
**VTQ57351.1**	*Campylobacter jejuni*	Epsilonproteobacteria	Campylobacterales	*Campylobacteraceae*
**WP_026741805.1**	*Lonsdalea quercina*	Gammaproteobacteria	Enterobacterales	*Pectobacteriaceae*
**WP_029593062.1**	*Siccibacter turicensis*	Gammaproteobacteria	Enterobacterales	*Enterobacteriaceae*
**WP_101348641.1**	*Escherichia coli*	Gammaproteobacteria	Enterobacterales	*Enterobacteriaceae*
**WP_194445129.1**	*Klebsiella pneumoniae*	Gammaproteobacteria	Enterobacterales	*Enterobacteriaceae*
**WP_062456638.1**	*Vibrio shilonii*	Gammaproteobacteria	Vibrionales	*Vibrionaceae*
**WP_086940253.1**	*Thaumasiovibrio occultus*	Gammaproteobacteria	Vibrionales	*Vibrionaceae*
**WP_008485098.1**	*Gallaecimonas xiamenensis*	Gammaproteobacteria	*incertae sedis*	-
**WP_087036954.1**	*Oceanisphaera profunda*	Gammaproteobacteria	Aeromonadales	*Aeromonadaceae*
**TXH95157.1**	*Pseudorhodobacter sp.*	Alphaproteobacteria	Rhodobacterales	*Rhodobacteraceae*
**WP_028109905.1**	*Ferrimonas futtsuensis*	Gammaproteobacteria	Alteromonadales	*Ferrimonadaceae*
**WP_031567826.1**	*Pararheinheimera texasensis*	Gammaproteobacteria	Chromatiales	*Chromatiaceae*
**WP_011073304.1**	*Shewanella oneidensis*	Gammaproteobacteria	Alteromonadales	*Shewanellaceae*
**WP_011495637.1**	*Shewanella denitrificans*	Gammaproteobacteria	Alteromonadales	*Shewanellaceae*
**WP_089349171.1**	*Pseudoalteromonas espejiana*	Gammaproteobacteria	Alteromonadales	*Pseudoalteromonadaceae*
**WP_018692378.1**	*Algicola sagamiensis*	Gammaproteobacteria	Alteromonadales	*Pseudoalteromonadaceae*
**WP_054581066.1**	*Acinetobacter equi*	Gammaproteobacteria	Pseudomonadales	*Moraxellaceae*
**WP_016657436.1**	*Acinetobacter rudis*	Gammaproteobacteria	Pseudomonadales	*Moraxellaceae*
**WP_004924459.1**	*Acinetobacter baylyi*	Gammaproteobacteria	Pseudomonadales	*Moraxellaceae*
**WP_004854861.1**	*Acinetobacter gerneri*	Gammaproteobacteria	Pseudomonadales	*Moraxellaceae*

**WP_134244962.1**	*Alkanindiges illinoisensis*	Gammaproteobacteria	Pseudomonadales	*Moraxellaceae*
**WP_066804633.1**	*Moraxella oblonga*	Gammaproteobacteria	Pseudomonadales	*Moraxellaceae*
**STY87967.1**	*Moraxella ovis*	Gammaproteobacteria	Pseudomonadales	*Moraxellaceae*
**WP_078307184.1**	*Moraxella lincolnii*	Gammaproteobacteria	Pseudomonadales	*Moraxellaceae*
**WP_028858284.1**	*Psychrobacter phenylpyruvicus*	Gammaproteobacteria	Pseudomonadales	*Moraxellaceae*
**WP_011279654.1**	*Psychrobacter arcticus*	Gammaproteobacteria	Pseudomonadales	*Moraxellaceae*
**WP_093067772.1**	*Psychrobacter pacificensis*	Gammaproteobacteria	Pseudomonadales	*Moraxellaceae*
